# RNAi Screening in Primary Human Hepatocytes of Genes Implicated in Genome-Wide Association Studies for Roles in Type 2 Diabetes Identifies Roles for CAMK1D and CDKAL1, among Others, in Hepatic Glucose Regulation

**DOI:** 10.1371/journal.pone.0064946

**Published:** 2013-06-20

**Authors:** Steven Haney, Juan Zhao, Shiwani Tiwari, Kurt Eng, Lin T. Guey, Eric Tien

**Affiliations:** 1 Target Generation Unit, Pfizer Research Technology Center, Cambridge, Massachusetts, United States of America; 2 Oligonucleotide Therapeutics Unit, Pfizer Research Technology Center, Cambridge, Massachusetts, United States of America; University of Arkansas for Medical Sciences, United States of America

## Abstract

Genome-wide association (GWA) studies have described a large number of new candidate genes that contribute to of Type 2 Diabetes (T2D). In some cases, small clusters of genes are implicated, rather than a single gene, and in all cases, the genetic contribution is not defined through the effects on a specific organ, such as the pancreas or liver. There is a significant need to develop and use human cell-based models to examine the effects these genes may have on glucose regulation. We describe the development of a primary human hepatocyte model that adjusts glucose disposition according to hormonal signals. This model was used to determine whether candidate genes identified in GWA studies regulate hepatic glucose disposition through siRNAs corresponding to the list of identified genes. We find that several genes affect the storage of glucose as glycogen (glycolytic response) and/or affect the utilization of pyruvate, the critical step in gluconeogenesis. Of the genes that affect both of these processes, CAMK1D, TSPAN8 and KIF11 affect the localization of a mediator of both gluconeogenesis and glycolysis regulation, CRTC2, to the nucleus in response to glucagon. In addition, the gene CDKAL1 was observed to affect glycogen storage, and molecular experiments using mutant forms of CDK5, a putative target of CDKAL1, in HepG2 cells show that this is mediated by coordinate regulation of CDK5 and PKA on MEK, which ultimately regulates the phosphorylation of ribosomal protein S6, a critical step in the insulin signaling pathway.

## Introduction

The incidence of Type 2 diabetes is roughly 10% of adults in the Western cultures and is expected to double or triple by 2050 [1]. It is rising quickly in Asian and underdeveloped regions of the world as they adopt an increasingly Western diet and lifestyle. Diabetes is strictly defined as a dysfunction in the regulation of glucose levels in the blood through impaired fasting glucose (IFG, measured after an 8-hour fasting), impaired glucose regulation (IGR, which is measured after fasting and then 2 hours following ingesting 70 g of glucose), or high levels of glycosylated hemoglobin (which results from high serum glucose levels). Diabetes can be managed to some extent by several well-established drugs, but many people do not show improvement using available therapeutics, and given the rising disease burden of diabetes, even small segments of patients that would benefit from one or more new therapeutic strategies could represent large patient populations. Diabetes is one of several chronic illnesses where the expansion of therapeutic options to include antibodies has followed from the increases in disease incidence and the recognition of the economic and personal impact the inability to treat them effectively. Current examples include the clinical development of Atorvastatin (anti-PCSK9) for the treatment of hypercholesterolemia [2]and Gevokizumab (anti-Il-1β) for type 2 diabetes [3], as well as the preclinical advancement of antibodies targeting FGFR1 [4], the insulin receptor [5] and the glucagon receptor [6] for type 2 diabetes.

The most common strategies for treating diabetes is through (a) increasing insulin levels, either through supplementing insulin directly or the use of drugs that increase insulin production by the pancreatic beta-cells, such as sulfonureas, and incretins, and (b) increasing insulin responsiveness in the liver and skeletal muscle, such as with metformin, despite an appreciation of mechanistic distinctions within the diabetic population, treating diabetes is difficult because of significant and varied co-morbidities, such as obesity, cardiovascular disease and renal failure. In many cases, these co-morbidities can influence the treatment strategy more than the specific manifestation of glucose and insulin dysfunction, further complicating treatment options.

The complex nature of the genetic contribution to diabetes incidence has been well appreciated, but in recent years, methods for characterizing this contribution has helped clarify matters. In particular, our understanding of diabetes genetics has been expanded in the last few years through the publication of several genome-wide association studies, GWAS [7]–[10]. In some cases, these loci are linked to genes previously identified as important to the onset of diabetes, such as TCF7L2, PPARG and GCK, which confirm the appropriateness of the approach, however, these studies have also added dozens of new candidate genes to the list of genetic factors that contribute to the onset of Type 2 Diabetes. While valuable in describing this genetic framework for understanding diabetes, the studies only partially explain how genetics contributes to glucose metabolism and diabetes. Two important reasons for this are intrinsic to the nature of GWA studies. First, these studies identify genetic polymorphisms that differ statistically in disease populations. These single nucleotide polymorphisms (SNPs) are sentinel mutations, and typically reside in non-protein coding regions of the genome. As such, they do not identify causative mutations, but are hypothesized to be linked to one or more causative mutations. Second, these sentinel polymorphisms identify small regions of the genome that have low recombination frequencies and are bracketed by segments of heightened recombination frequencies, defining blocks or clusters of linked genes. Genes within these regions are said to be in linkage disequilibrium because they recombine as a block of genes. As such, a SNP that shows a biased frequency in a disease population implicates one or more candidate genes, rather than a single gene. Another important reason why GWA studies alone have not been able to describe the mechanism of glucose dysregulation is that many of these candidate genes are novel, suggesting that they either impinge on glucose regulatory pathways in ways that have not been characterized previously or define new pathways. In order to determine which genes do play causative roles in diabetes, and to frame those genes into biochemical pathways, complementary methods for characterizing these candidate genes is required.

RNAi screening has been very successful for establishing the function of genes at the candidate, gene family and whole genome levels [11], [12], but its utility in defining function is dependent on the extent to which the model being screened actually represents the biological question. For example, CDKN2A and CDKN2B have been identified in diabetes GWA studies, suggesting that cell cycle arrest or some other unknown function of these genes plays a role in Type 2 diabetes. Since the expression of these genes is lost in immortalized and transformed cell lines, such systems would be ineffective to determine the role of genes such as CDKN2A in diabetes, as well as any gene that interacts with this role. A primary cell model would be best suited for such studies, but systems are complex. Primary hepatocytes and human islets are isolated from post-mortem donors (introducing significant inter-experimental variability), while primary skeletal muscle and adipocytes are frequently generated from mesenchymal stem cells through processes which are frequently incomplete, leaving 30% or more of the cells in incompletely differentiated states. Finally, maintaining primary cells generally requires great care, and stress can lead to dedifferentiation. Despite the challenges, primary cell systems present the broadest opportunity to define the function of a novel gene in diabetes.

We have undertaken a study of the role of many genes identified in the recent GWA studies of Type 2 diabetes [8]–[10], [13] in primary human hepatocytes. The liver plays critical roles in glucose regulation, including the absorption of glucose from the blood, conversion to glycogen when levels are high, and the generation of glucose from glycogen breakdown as well as gluconeogenesis from lactate and pyruvate when systemic glucose levels are low. Dysregulation of these processes occur in insulin resistance, pre-diabetes (both IFG and IGT) and frank diabetes. As such, the likelihood that one or more of the genes encoded within a loci implicated in GWAS would be involved in the regulation of glucose metabolism in the liver is high. The reduced ability to store serum glucose as glycogen and the failure to repress gluconeogenesis when serum glucose levels are high are both clinically relevant examples of hepatic glucose dysregulation that are observed in diabetes. In these studies, we have identified multiple genes, including CAMK1D, CDKAL1, TSPAN8 and KIF11, whose implied role in diabetes from the GWA studies was further supported by effects seen on gluconeogenic and glyconeogenic pathways in primary human hepatocyte cultures. These genes represent novel pathways and targets for potential therapeutic intervention in the treatment of Type 2 diabetes.

## Materials and Methods

### Primary hepatocyte cell culture and siRNA transfection

Williams E and DMEM media, selenium, FBS, trichostatin A, dexamethasone, BSA, glutamine, penicillin/streptomycin, glucose, Periodic-Acid Schiff staining reagent kits, were from Sigma-Aldrich (St. Louis, MO). Insulin, and tranferrin were from Lonza (Rockland, ME). siGenome and On-Target Plus siRNA Smartpools used for screening were from Dharmacon/Thermo (Lafayette, CO). Additional siRNAs used in validation experiments were from Ambion/Applied Biosystems (Austin, TX).

Culture of primary human hepatocytes has been previously described [14]. Briefly, primary human hepatocytes were seeded onto tissue culture plates pretreated with rat tail collagen (BD Biosciences, San Jose, CA) in DMEM plating medium. The following morning, growth factor reduced Matrigel (BD Biosciences) was overlaid onto the cells. For the primary screens, 25 µg/well of Matrigel™ was used, for immunofluorescence studies, 19 µg/well was used. For screening of glycogen levels and for immunofluorescence studies, insulin levels were reduced to 40 pM, to allow for insulin sensitivity. Transferrin and selenium were added individually. Experiments were performed in three separate lots of primary hepatocytes, Hu4000, Hu4161 (CellzDirect/Life Technologies) and ZBH220 (ZenBio, Research Triangle Park, NC).

### Commercial siRNAs and transfection

siRNAs used in the primary screen were obtained from Dharmacon (Dharmacon/Thermo Scientific). siGenome and On-Target Plus siRNAs for each of the genes listed in **Table 1** were used at 50 nM after formation of transfection particles with RNAiMAX (Invitrogen/Life Technologies, Carlsbad, CA). Additional genes, intended as positive and negative controls were screened as well, in this case as siGenome siRNA Smartpools only. Knockdown of target genes were verified by RT-PCR.

**Table 1:** Statistically significant SNPs identified in GWA studies and linked genes.

Chromosome	SNPs associated with Type 2 Diabetes	Candidate genes	AKA	Function	Notes
1	rs10923931	NOTCH2		Jagged receptor, pancreatic differentiation	beta cell; pancreatic differentiation
2	rs1801262	NEUROD1	MODY6, BETA2	beta-cell transactivator A2, insulin expression	beta cell; insulin expression
	rs3772267, rs3842570, rs5030952	CAPN10		protease	ubiquitious expression; insulin secretion or hepatic glucose transport
	rs7578597	THADA		rearranged in thyroid cancers, high expression in liver	hepatocytes;
3	rs1801282	PPARG		transcription factor, interacts with RXR family, adipocyte differentiation	adipocytes and hepatocytes; adipocyte differentiation and role in hepatic steatosis
	rs5400	SLC2A2	GLUT2	glucose transporter	hepatocytes and beta-cells; glucose transport
	rs4402960, rs1470579	IGF2BP2		pancreatic development, IGF expression	beta cells; circulating IGF levels
	rs4607103	ADAMTS9		metalloprotease	wide expression; cell type differentiation
4	rs10010131, rs1801214	WFS1		Wolfram Syndrome, b-cell and brain expression, ER protein	beta cell;
6	rs7754840, rs10440833	CDKAL1		pancreatic and skeltal muscle, affects CDK5/CDK5R1	beta cells, skeletal muscle; reduced beta cell function, glucose sensitivity
7	rs1799884	GCK	MODY2	glucokinase	hepatocyte, skeletal muscle; insulin response to increase glucose uptake
	rs864745, rsa849134	JAZF1	TIP27	Zinc finger protein	rs864745 SNP accociated with reduced glucose response
	rs12531767, rs1260589	EXOC4	SEC8, REC8	exocyst complex component 4	associated SNPS and deletions correlated with glucose levels
		LRGUK		guanylate kinase	associated SNPS and deletions correlated with glucose levels
8	rs13266634, rs7923837, rs3802177	SLC30A8		b-cell zinc transporter, insulin production	beta cells; T1D autoantigen, associated SNP shows reduced insulin expresion
9	rs10811661, rs10965250	CDKN2B	p15, INK4B, ARF	inhibits CDK4 and CDK6,	
		CDKN2A	p16, INK4A	inhibits CDK4	
10	rs7903146, rs12255372, rs7901695	TCF7L2	TCF4	b-catenin TF, insulin secretion	adipocytes; insulin secretion and glucose sensitivity
	rs1111875	HHEX	PRHX, PRH	homeobox	hepatocytes, beta, hematopoietic and endothelial cells; hepatocyte differentiation and insulin glucose sensitivity
		KIF11	EG5	kinesin, target of monastrol	
		IDE		insulin degrading enzyme, b-amyloid function in AD identified	
	rs12779790	CDC123	D123, C10orf7	cell-cycle (G1) regulation	rs12779790 SNP associated with reduced glucose response
		CAMK1D		calmodulin-dependent kinase, responds to IL and IL8, activates ERK1	rs12779790 SNP associated with reduced glucose response
11	rs5219, rs5215	KCNJ11	Kir6.2	inwardly rectifying potassium channel, beta-cell expression	beta cell; insulin secretion
		ABCC8	SUR1	sulfonylurea receptor	beta-cells; potassium transporter linked to insulin secretion
	rs7480010	LOC387761		unknown	
	rs1113132	EXT2		exostosin 2, Golgi resident secretion	
		ALX4	FPP, PFM	apolioprotein, aristaless-like homeobox 4	
12	rs7957197	HNF1A	MODY3, TCF1	hepatocyte nuclear factor 1A,	hepatocyte and beta-cell differentiation,
	rs7961581	TSPAN8		ovarian cancer antigen/antibody therapeutic target	rs7961581 SNP accociated with reduced glucose response
		LGR5	GPR49	stem cell antigen, GPCR	skeletal muscle, other tissues;
	rs62871062	IAPP		islet amyloid polypeptide	beta-cells; islet function
13	rs9551419	IPF1	MODY4, PDX1, IDX1	insulin promoter factor 1, pancreatic	insulin expression
16	rs8050136, rs9939609, rs11642841	FTO		Fat Mass and Obesity Related Gene, oxoglutarate-dependent oxygenase	
17	rs757210, rs4430796	TCF2	MODY5, HNF1B	hepatocyte nuclear factor 1B,	hepatocyte and beta-cell differentiation,
20	rs3212183	HNF4A	MODY1	hepatocyte nuclear factor 4A	hepatocyte and beta-cell differentiation,

### Lentiviral vectors

Lentiviral constructs of CAMK1D and CDK5 were synthesized by GeneArt (Toronto, Canada), and cloned into the pLVX-puro vector (BD Clontech, Mountain View, CA). Infective virus was prepared using the Lenti-X HT packaging kit (BD Clontech), according to manufacturer's instructions. All genes, including those expressing mutant kinases were synthesized *de novo*.

### PAS assay for glycogen levels

Periodic Acid-Schiff staining for cellular glycogen was performed as directed by the manufacturer (Sigma-Aldrich). For fluorescence quantification of PAS staining levels, cells were stained with DAPI for 10 minutes immediately before imaging the cells.

### Measurement of RNA levels using real-time PCR

RT-PCR was used to measure mRNA expression levels using the Taqman reverse transcriptase platform from Applied Biosystems (Foster City, CA). Probes were designed for each gene by the manufacturer and RNA from primary hepatocytes was prepared and used according to the manufacturer's protocols.

### Measurement of pyruvate utilization

Primary human hepatocytes were plated as described above and allowed to culture for 2 days prior to assay with daily media changes. On Day 3 post plating, media was changed to Pyruvate Media which is based on standard culture media described in [14] with the following alterations: DMEM, glucose free, lactate free, pyruvate free, phenol red free based medium (Sigma, St Louis, MO), 5 mM glucose, 500 uM pyruvate, 40 pM human recombinant insulin. On Day 4 post plating, media was removed and cells were very gently inverted on to a sterile absorbent pad to remove residual media without disturbing Matrigel™ overlay. Fresh media with or without 1 nM human recombinant insulin was immediately added to the wells and cells were placed back at 37C. After 24 hours (Day 5 post plating), pyruvate was measured in the media using Pyruvate Assay Kit (Biovision, Mountain View, CA) according to manufacturer protocol for colorimetric absorbance assay.

### Indirect immunofluorescence

Primary antibodies against CRTC2 were obtained from Santa Cruz Biotechnologies (Santa Cruz, CA), phosphorylated CREB^S133^ were obtained from both Santa Cruz Biotechnologies and Cell Signaling Technologies (Beverly, MA), and phosphorylated ribomosomal protein S6 were obtained from Cell Signaling Technologies. Results for CRTC2 and phosphorylated CREB were observed with three different primary antibodies. Secondary antibodies and DAPI were obtained from Molecular Probes/Life Technologies (Eugene, OR). Cells were fixed for 10 min with 2% paraformaldehyde (Alfa Aesar, Ward Hill, MA). Cells were permeabilized with 0.2% Triton X-100 (Sigma-Aldrich, St. Louis, MO) for 10 minutes, and blocked in blocking buffer (5% donkey serum; Jackson ImmunoResearch Labs, West Grove, PA) in 0.2% Triton X-100, from 1 hr to overnight. Washing steps were performed using PBS. Primary antibodies were added to wells for 1–3 hr in blocking buffer with occasional agitation, and plates were washed with PBS three times. Secondary antibodies were Alexa-labeled donkey antibodies (Life Technologies) added at a 1∶200 dilution for one hour, along with DAPI. Images were captured on a Thermo/Cellomics VTi (Pittsburgh, PA) and images were analyzed using CellProfiler, an open-source image analysis platform [15].

### Inhibition of protein kinases

Small molecule inhibitors were used as described in the text following their resuspension in anhydrous DMSO (Sigma-Aldrich) at concentrations 1000 times that of the final concentrations, with addition to cells made as 20× solutions in William's E medium base. The protein kinase A inhibitor H89 and the KIF11/Eg5 kinesin inhibitor monastrol were purchased from Sigma, the RSK inhibitor BI-D1870 was purchased from B-Bridge International (Cupertino, CA) and the PKC inhibitor bis-indolylmaleimide (BIM) was purchased from EMD-Millipore Chemicals (Bilerica, MA).

### Statistical analysis

Image features extracted from CellProfiler [15] were analyzed at the cellular-level. Silencing of targeted genes were compared to non-targeting siRNAs (including siGenome non-targeting siRNAs from Thermo/Dharmacon) in glycogen and pyruvate assays with Student's t-test.

## Results

### An RNAi screen for genes that effect glucose regulation and disposition in the liver

As discussed above, a cellular model contributes to our understanding of the genetic basis of disease only if it recapitulates the functions of the tissue or cell type being studied. Only in such cases would an *in vitro* observation help to explain a genetic change. In considering the hepatic functions relevant to diabetes, there are two central roles played by the liver in glucose regulation. First is the post-prandial absorption of glucose following a meal, which acts as an important buffer for serum glucose levels. Diabetics have a diminished capacity to store glucose as glycogen [16]. Second, insulin resistance also increases hepatic gluconeogenesis despite high serum glucose levels [17]. Hepatic glucose production is controlled by the expression of PEPCK, an enzyme that converts pyruvate to oxalo-acetic acid and is the rate limiting step in gluconeogenesis. Expression of the PEPCK gene, PCK1, is controlled by insulin, through the nuclear exclusion of the FOXO1a transcription factor [18], and by glucagon, through the expression of the PPARμ-cofactor PGC-1α, whose expression is in turn regulated through CREB and CRTC2 [19] [20].

We defined three challenges to developing a cell-based experimental system that appropriately models glucose metabolism. The first was a cell culture model for liver function. Seventy percent of the liver is comprised of hepatocytes, and they mediate glucose disposition directly. Widely used models for hepatocytes, such as the HepG2 cell line are capable of regulating gene expression through the insulin pathway (a critical aspect of glucose regulation), but do not form cell-cell junctions or bile canaliculi to the same extent as primary hepatocytes. These structural features are essential for some regulatory functions in hepatocytes. Given the number of highly novel and uncharacterized genes that have recently become implicated to the genesis of diabetes, a model system that recapitulates a greater number of hepatocellular functions will provide a more complete assessment of gene function. Primary human hepatocytes are used in toxicological studies because *in vitro* culture methods have been developed that preserve important tissue-level interactions between cells, such as bile canaliculi. Cells cultured under such systems show more *in vivo*-like hepatic function, such as the generation of albumin and urea, and maintain a normal pattern of expression of P450 enzymes for a longer period of time [21]. Such models are important for toxicology because many compounds are toxic for unrelated reasons unrelated to their desired mechanism of action [14]. While the primary human hepatocyte cell culture systems are technically complicated and significantly more expensive than maintaining cell lines, the value of a cell culture system capable of detecting toxic responses is critical.

**Figure 1:**
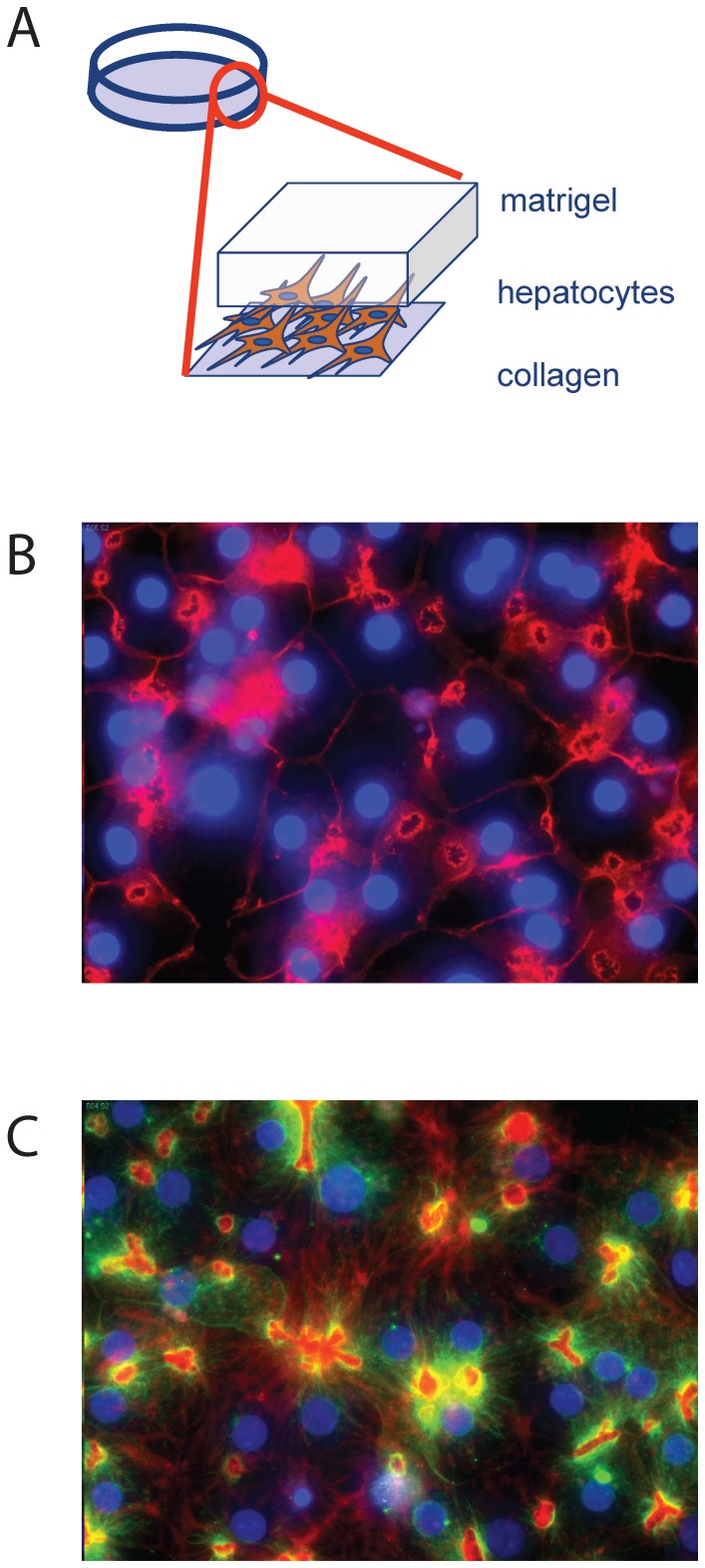
The hepatocyte cell culture system and the measurement of perturbations to hepatic glucose regulation. A. Diagram of the *in vitro* hepatocyte cell culture system. Primary hepatocytes are plated on an α 1-collagen matrix at a high density. 24 hr later, growth-factor reduced matrigel is added to the culture. B. Indirect immunofluorescence of primary hepatocytes showing nuclei in blue and β-catenin in red. β-catenin lines the cell-cell junctions and highlights the bile canaliculi, deposits of bile acids produced by hepatocytes. C. Indirect immunofluorescence of hepatoctytes stained for nuclei (blue), β-tubulin (green) and actin (red).

A collagen-Matrigel™ sandwich culture of primary human hepatocytes could provide insight to potential hepatic roles for the candidate genes (**Figure 1A**). We were able to modify the original system [22], particularly lowering the high levels of insulin used in media, to produce cells that still formed bile canaliculi (**Figure 1B, 1C**), were responsive to insulin, secreted urea and albumin. We were also able to show that these cells could be transfected with siRNAs readily, using several commercially available lipid reagents. The bile canaliculi are an example of the importance of the cell system in novel gene function studies. The production and management of bile acids is critical to the function of human hepatocytes and the liver, in large part because of their effects on signaling within hepatocytes, including the insulin and glucagon signaling pathways [23], [24]. As shown in **Figure 1B** and **Figure 1C**, hepatocytes invest significant energy into forming bile canaliculi.

**Figure 2:**
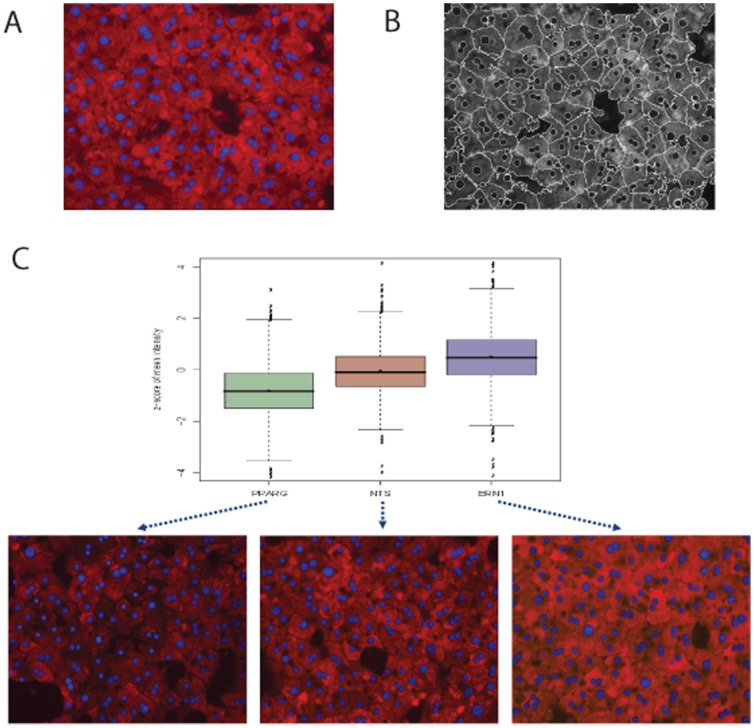
Quantitative fluorescence imaging of hepatocyte glycogen levels. A. Fluorescent image of PAS-stained hepatocytes. Staining levels reflect glycogen content. B. Image analysis of PAS-stained hepatocytes. C Quantification of glycogen levels of siRNA-treated hepatocytes by image analysis. Hepatocytes were treated with insulin for 1 hr in medium with a physiological glucose concentration.

Methods exist for measuring glycogen levels, including histological techniques. Insulin signals hepatocytes to increase glucose transport convert the glucose to glycogen; hepatic glycogen levels are reduced in patients with insulin resistance and diabetes [25], [26]. One such method for measuring glycogen levels, Periodic Acid-Schiff staining (PAS), is a histological method that detects glycogen through the formation of red pigments that form adducts to glycogen. These adducts are also highly fluorescent, enabling imaging approaches to the quantification of glycogen levels, as shown in **Figure 2A**. We explored the use of quantitative imaging of PAS-fluorescence in pilot studies, including the ability of the method to detect the treatment of cells with insulin and glucose, and effects of siRNAs in perturbing these responses. Gross staining of fixed cells confirmed the reliability of the method to detect increased glycogen levels (**Figure S1**). Hepatocytes were plated on imaging microtiter plates and scanned at wavelengths in the red-orange range (546, 555, 568, 594 and 647 wavelengths). PAS-adducts are broadly fluorescent and all of these wavelengths accurately captured the differences in staining levels between samples. Images of the hepatocytes were analyzed using CellProfiler [15] to segment (**Figure 2B**) and quantify intensity levels. Further analysis at the well, field and cell level of the images were all effective methods for quantifying glycogen levels (Z′ = 0.39 using field-level data comparing 1 nM insulin to no insulin as positive and negative controls), as shown in **Figure S1**. For the siRNA screen, differences in glycogen accumulation were measured as changes in fluorescence intensity of hepatocytes per field, across several fields per well, typically 10. The image analysis was able to quantify changes in glycogen levels in hepatocytes treated with siRNAs, as shown in **Figure 2C**. siRNAs against PPARG and ERN1 were able to lower and raise glycogen levels, relative to a non-targeting control (NTS), as determined both visually, in the standard histological analysis of PAS staining as a measure of glycogen levels in hepatocytes, and by quantitative image analysis.

**Figure 3:**
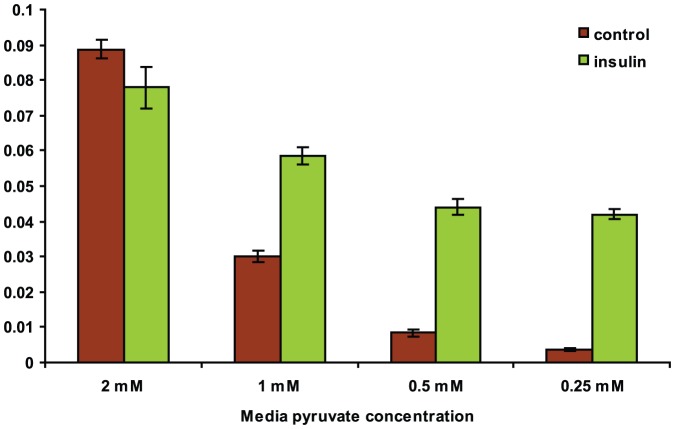
Media pyruvate levels are dependent on gluconeogenic activity of primary hepatocytes. Measurement of pyruvate levels in hepatocyte cell culture media following insulin treatment. Sensitivity of the assay is increased as exogenous pyruvate levels are reduced.

To measure gluconeogenesis, we adapted standard pyruvate utilization assays for the hepatocyte system. Under conditions of low glucose, the liver will synthesize and secrete glucose for use by the peripheral tissues. Intermediates such as pyruvate are substrates for this process in the liver, and *in vitro*, pyruvate utilization can be measured through its depletion from the media, since the bulk of pyruvate utilization in the fasting state is through gluconeogenesis Hepatocytes treated with 1 nM human recombinant insulin show a significant decrease in pyruvate utilization as evidence by higher levels of pyruvate measured in the media after treatment (**Figure 3**). siRNAs targeting the insulin receptor were used to verify that the method was sensitive to RNAi knockdown treatments (data not shown).

siRNAs targeting the replicated T2D genes from several studies [9], [10], [13] were used to evaluate potential roles in hepatic glucose regulation. The genes screened in this study are listed in **Table 1**. For each of these genes, two siRNA smartpools were used to test the function of each gene on the glyconeogenesis and gluconeogenesis endpoints. Concordance between the two siRNAs Smartpools for the glyconeogenesis endpoint was high, with an intraclass correlation coefficient of 0.87 (95% CI: 0.73–0.93). In addition, several genes were used as controls for this study, including genes affecting ER stress, which is directly related to diabetes and additional standard positive and negative controls [27], [28], detailed in **Table S1**. These genes were used to characterize general cell stress pathways on hepatic glucose regulation because it was unclear whether the candidate genes would affect glucose regulation directly or whether they would induce a stress response that could have an effect on glucose regulation indirectly. This was a new experimental system, so the actual performance these controls were speculative, particularly the induction of apoptosis by mitotic inhibition, as the majority of primary hepatocytes are postmitotic. However, the inclusion of as many cell stress and death responses would be helpful for deconvoluting the cellular responses to the target genes, and this panel was a prototype for a family of cellular diabetes models, including pancreatic b-cells, skeletal muscle, adipocytes, inflammatory cells and the endothelium.

**Figure 4:**
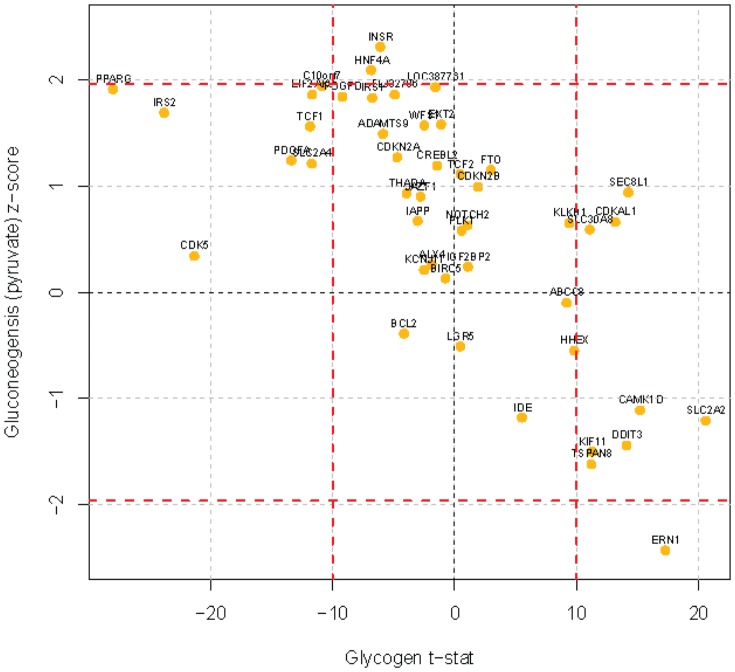
Effect of siRNAs on hepatocyte glucose regulation. siRNAs corresponding to the T2D-replicated GWA loci were transfected into primary hepatocytes. The effects on glycogen accumulation and pyruvate utilization were measured in separate assays.

The assay scores for each gene are plotted in **Figure 4**. The data shows how each siRNA affected the two endpoints, glycogenesis along the X-axis, where increased glycogen deposition is therapeutically beneficial, and gluconeogenesis, where decreased glucose generation during the high insulin/glucose state is desired. According to these criteria, genes at the lower right quadrant are those that score most favorably. In addition, the effect of these siRNAs in reducing expression of the genes they target was also measured. We used RT-PCR to verify target gene silencing. The data are presented in **Table S2** as the percentage of knockdown of the target gene by each of the two pools for that gene. Several genes could not be detected by RT-PCR, including ABCC8, ADAMTS9, IPF1 and SLC30A8. Roles for these genes in glucose regulation have already been described for other tissues, particularly IPF1 and SLC30A8, but (as noted above) the panel was developed to be a comprehensive test of the candidate genes in all tissue systems relevant to glucose regulation and diabetes.

The glycogen imaging assay was intrinsically more robust than the pyruvate depletion assay, so 9 genes scored as statistically significant in the glycogen assay, while only one gene, the ER stress control gene ERN1 scored as statistically significant in the pyruvate assay. However, the two assays were able to identify a strong trend in the target gene panel and the trend was supported by the known biological roles of the control genes that affect glucose regulation in the liver, so data was sufficient to identify a subset of target genes that could be validated in subsequent experiments. Specifically, two of the six genes showing an increase in glycogen storage and a decrease in gluconeogenesis could be readily linked to roles in human hepatic glucose metabolism based on published reports. SLC2A2 (aka GLUT2) is the major glucose transporter for glucose out of hepatocytes. Suppressing its function may trap glucose in the cell, leading to formation of glycogen as a means of storing glucose more effectively. This hypothesis is supported by clinical evidence showing mutations in SLC2A2 reduce transporter function cause Fanconi-Bickel Syndrome [29], [30], which leads to an enlarged liver resulting from excessive glycogen accumulation [31]. ERN1 (also known as IRE1) is an ER transmembrane kinase that responds to significant ER stress and can induce apoptosis [32]. The role of ER stress in diabetes has been well established [27], and specific roles in liver, adipose and skeletal muscle insulin resistance have also been described [33], including the inhibition of CRTC2, which results in hepatic insulin resistance in mice [28]. An increase in insulin sensitivity and a reduction of ER stress has been observed in obese patients following gastric bypass surgery, suggesting a link in clinical metabolic syndrome [34].

Several genes associated with diabetes, but with currently uncharacterized roles in glucose regulation were also identified. TSPAN8 is a member of a gene family of integral membrane proteins which has been shown to play important roles in cell signaling. Tetraspanins associate in hetero and homo-oligomers to create membrane microenvironments which then coordinate the formation of signaling complexes, including G-proteins and intracellular protein kinases [35]. A well characterized gene that shows a novel phenotype in this assay is KIF11. This kinesin plays an essential role in mitosis and its inhibition by siRNAs or the inhibitor Monasterol, is typically lethal to cancer cells [36]. Human primary hepatocytes are quiescent *in vivo* and in this culture system, but a non-mitotic role in microtubule-dependent processes (including organelle trafficking or vesicle secretion) could be implicated. A role for Kinesin 1 has been described for insulin secretion in pancreatic beta-cells [37] and a secretory function KIF11 in hepatocytes is a possibility. CAMK1D, a calcium/calmodulin regulated protein kinase, was also identified in this screen. Calcium signaling plays essential roles in glucagon signaling, and in ER stress, which is one potential cause of insulin resistance (as discussed above). Previous work has defined roles for various CAMK and CAMKK isoforms [38]–[41], particularly in the activation of CREB and in signaling to AMPK, but no role for CAMK1D has been described.

### Knockdown of CAMK1D suppresses expression of PCK1 gene expression

**Figure 5:**
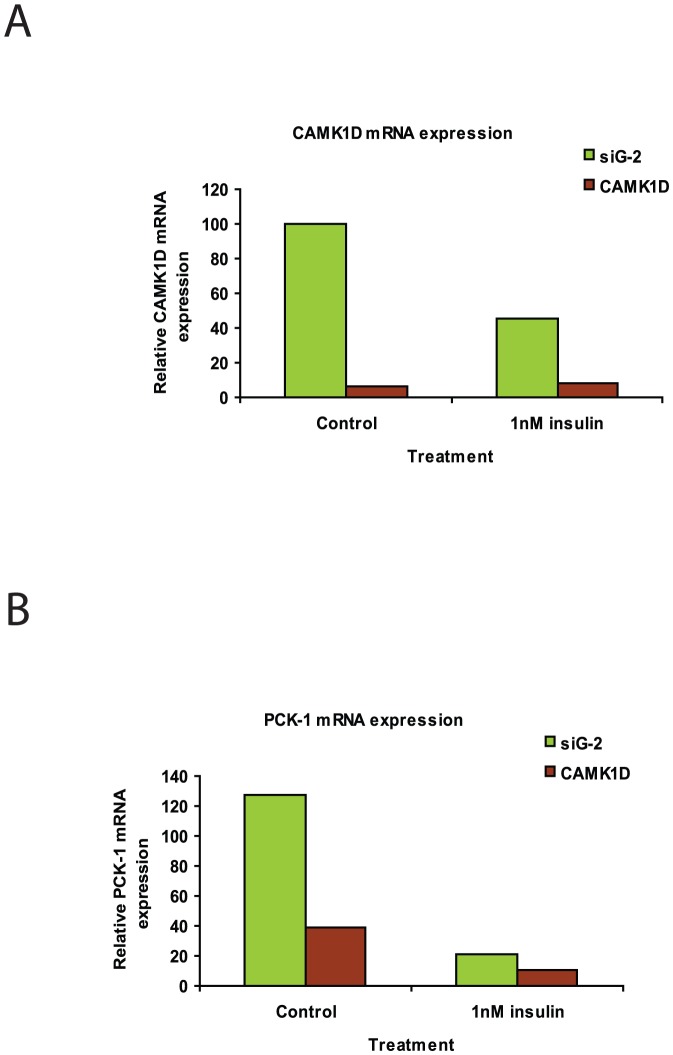
Effect of CAMK1D siRNA treatment on the expression of CAMK1D and of PCK1. mRNA levels of CAMK1D and PCK1 were measured in hepatocytes treated with siRNAs against CAMK1D by RT-PCR. siRNA treatments and mRNA analyses were run in presence and absence of insulin. A. Expression levels of CAMK1D. B. Expression levels of PCK1. All PCRs were normalized to GAPDH expression.

As a first step in the characterization of CAMK1D in the regulation of gluconeogenesis, we examined the effect of CAMK1D expression on PEPCK (PCK1). PCK1 expression is dependent on the FOXO1a, and PGC1α transcription factors (as well as direct effects of CRTC2), which are regulated by the insulin and glucagon signaling pathways. We investigated whether CAMK1D could have an effect on PCK1 expression. Human hepatocytes were transfected with either the control or the CAMK1D siRNAs, and the expression levels of CAMK1D and PCK1 analyzed by RT-PCR. **Figure 5A** shows the reduction in CAMK1D levels in hepatocytes transfected with targeted siRNAs. The knockdown is observed in both insulin treated and untreated hepatocytes. In **Figure 5B**, after CAMK1D knockdown, PCK1 expression is inhibited in insulin treated and untreated hepatocytes, providing evidence that CAMK1D is involved in glucose regulation in primary human hepatocytes. Furthermore, the fact that a reduction in PCK1 expression is observed in hepatocytes that have not been treated with insulin suggests that CAMK1D functions on an insulin-independent signaling pathway.

### siRNAs against CAMK1D, TSPAN8 and KIF11 reduce glucagon signaling to CRTC2

**Figure 6:**
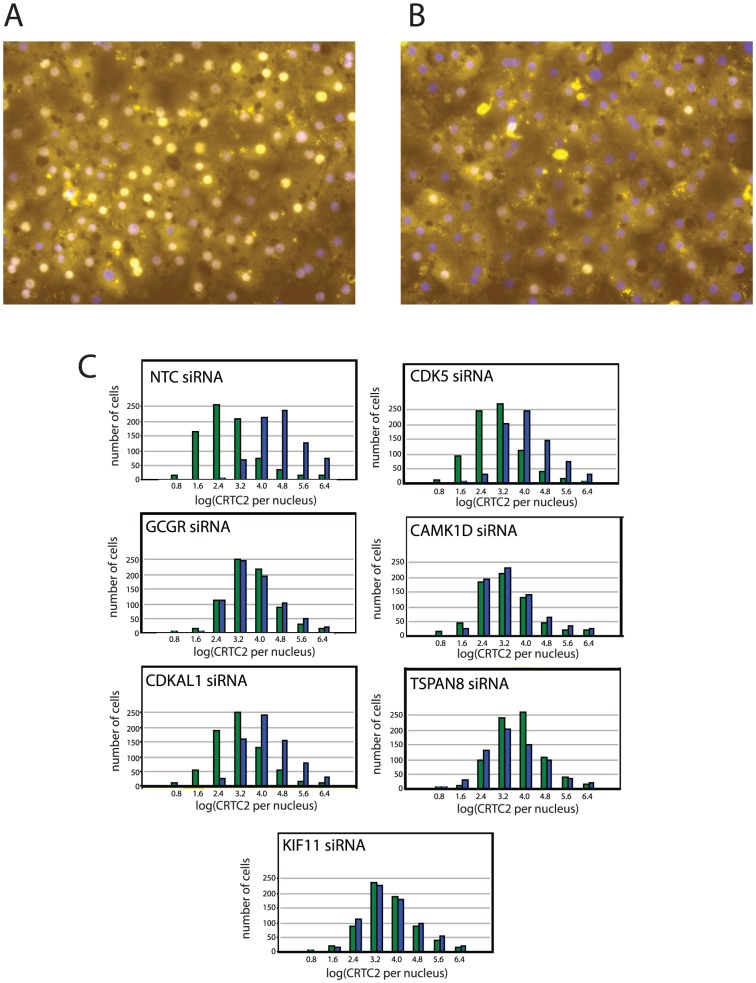
Effect of siRNAs that affect hepatic glucose regulation on glucagon-induced translocation of CRTC2. Primary hepatocytes treated with siRNAs for 48 hr and 100 nM glucagon for 2 hr. Images show staining of primary human hepatocytes with antibodies to exogenous CRTC2. Images are single fields of hepatocytes treated with (A) NTC (non-targeting control) or (B) GCGR (glucagon receptor) and glucagon treatment. Location of nuclei were determined by staining with DAPI and used to calculate the extent of CRTC1 localization to the nucleus. Nuclei are shown in blue and CRTC2 indirect immunofluorescence is shown in yellow. C. Effect of siRNAs on CRTC2 levels in the nucleus. Images were analyzed and scaled for CRTC2 staining levels in nuclei. Histograms that quantify the extent of CRTC2 nuclear localization for siRNA-treated cells are shown as labeled when treated with glucagon (blue bars) or untreated (green bars). Nuclear CRTC2 levels are in arbitrary units after transformation to a log scale.

Since the regulation of PCK1 by CAMK1D occurs independently of the insulin signaling pathway, its effects are less likely to occur through the localization of FOXO1α, which is regulated by insulin through the AKT protein kinase, and more likely to be mediated through the PPAR coactivator PGC-1 α. The expression of PGC-1 α is in turn regulated by CREB and the CREB-coactivator CRTC2. Of these two transcriptional cofactors, CRTC2 has been directly identified as critical to the glucagon response in the liver [42]. We looked at the effect of CAMK1D and other genes identified in this screen on the nuclear localization of CRTC2 following treatment of hepatocytes with glucagon. For these experiments we used antibodies to endogenous CRTC2, which allowed us to continue to work with primary human hepatocytes. The staining was weaker than what is observed in cell systems where CRTC2 is over-expressed or expressed as a fusion to GFP, but was still responsive to glucagon treatment (**Figure 6A and 6B**). CRTC2 staining in the primary hepatocytes was subtle, as primary hepatocytes are not amenable to generating stable expression of a fusion protein to GFP, so only endogenous protein was characterized, and primary hepatocytes have relatively high levels of autofluorescent pigments. Although we focused on CAMK1D in the RT-PCR experiment (above), as an initial experiment to investigate the biological function of the hits from this screen, we expanded our analysis of genes that may affect glucagon signaling to CRTC2 for this experiment. In addition to CAMK1D, we looked at the effects of TSPAN8 and KIF11, and CDKAL1, a gene that showed aneffect on the glycogen accumulation assay, and CDK5, the protein kinase that is negatively regulated by CDKAL1 and showed an opposite effect in the glycogen assay.

Primary hepatocytes were treated with siRNA for CAMK1D, CDKAL1, CDK5, TSPAN8, KIF11 and GCGR (the glucagon receptor). The effects on CRTC2 nuclear localization are shown in **Figure 6C**. When cells were treated with siRNAs against the glucagon receptor (GCGR), levels of nuclear CRTC2 did not increase. When siRNAs against CDKAL1 or CDK5 were transfected into hepatocytes, CRTC2 levels in nuclei did increase following stimulation with glucagon, indicating that these genes do not directly regulate the activation of this transcriptionsl cofactor. For CAMK1D, TSPAN8 and KIF11, siRNAs, CRTC2 localization to the nucleus was significantly blunted, Previous work has linked CAM kinases to calcium-medieated signaling and it has also been shown that activation of cAMP signaling is also required for CRTC2 activation. To determine whether CAMK1D plays a role in general cAMP signaling, we looked for an effect of CAMK1D siRNAs on the phosphorylation of ribosomal protein S6. cAMP activates PKA as well as Epac, and PKA activates the ERK/RSK, which phosphorylates the ribosomal protein S6 at residues 235 and 236. These sites are also phosphorylated by p70 S6 kinase, which is activated by insulin. In cells treated with CAMK1D siRNAs, phosphorylation of S6 at these residues was observed following treatment with both insulin and glucagon (results not shown), indicating that CAMK1D does not play a role in PKA/RSK signaling.

### CDK5 regulates PKA signaling through MEK/ERK kinases

Since CDKAL1 and CDK5 did not affect the expression of PCK1, the activation of CRTC2, or show an effect on gluconeogenesis in the initial siRNA screen, we performed some additional experiments to clarify their roles in hepatic glucose regulation. CDKAL1 has been described as a negative regulator of CDK5 through its homology to CDK5RAP1 [43], a well-characterized negative regulator of CDK5 [44] that functions through the inhibition of the CDK5 activator p35 [43], [45]. Recently, tRNA methyl transferase activites have been observed for both CDKAL1 and CDK5RAP1 [46], [47], suggesting additional complexities to the biology of CDK5, itself part of a complex that regulates selective mRNA transcripts through interactions with mRNA and tRNA synthetases [48], [49], further augmenting a large and diverse set of roles for CDK5 in multiple cell types [50].

Primary human hepatocytes are not amenable to genetic manipulation *in vitro*, as they are generally post-mitotic and will de-differentiate if handled according to normal clonal or transduction/selection methods. To make a connection between CDKAL1 and hepatic glucose regulation, we looked at CDK5 itself as a well-characterized protein that we could manipulate in an experimental system. These experiments were focused on the molecular mechanism of CDK5 and its effects on specific signaling pathways in liver cells in order to extend the observation of the effect of siRNAs against CDKAL1 and CDK5 in primary hepatocytes. We prepared transducing lentiviruses that expressed wild type CDK5, and several inactivating mutants: D144N inactivates the catalytic mechanism of the kinase, Y15F prevents the phosphorylation and activation of the kinase by upstream kinases including c-Abl, and K33T is also required for the phoshorylation of CDK5 [51]–[54] and transduced them into the hepatoma cell line HepG2.

**Figure 7:**
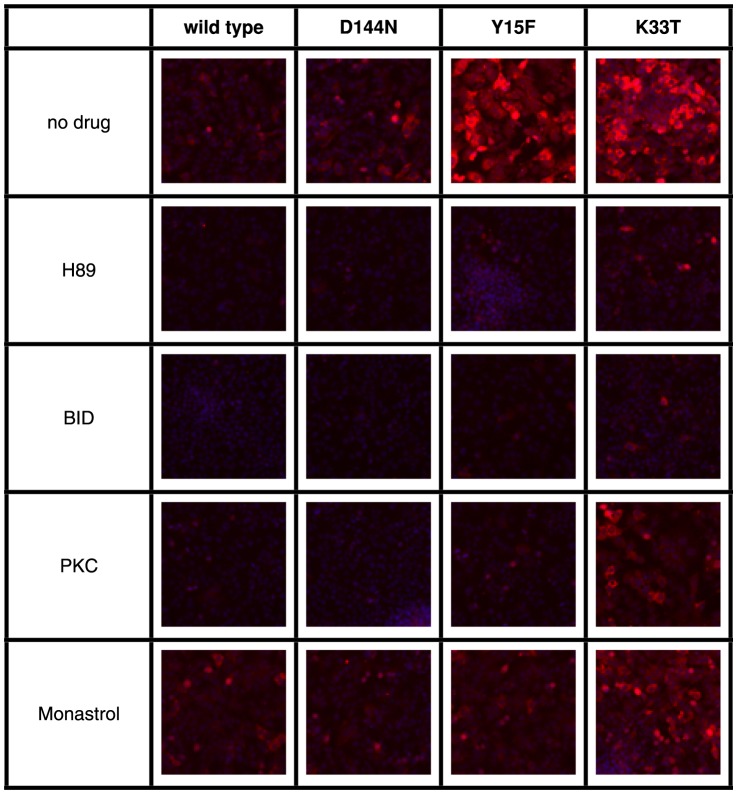
Effect of the expression of wild type and mutant forms of CDK5 on the phosphorylation of ribosomal protein S6. Effect of inhibitors of PKA PKC and RSK on RpS6 phosphorylation. Images are HepG2 cells tranduced with lentiviral vectors expressing forms of CDK5, as indicated at the top of each column. Cells were cultured in William's E medium without additional glucagon or insulin. Cultures from each line were treated with protein kinase inhibitors, as indicated by row. DNA is shown in blue and phosphorylated ribosomal protein S6 is shown in red. Treatment with inhibitors was for two hours prior to fixation and staining.

Results of expressing wild type CDK5 and the inactivating mutants discussed above in HepG2 cells are presented in **Figure 7**. The results show that transduction with the Y15F and K33T mutant forms of CDK5 result in increased phosphorylation of ribosomal protein S6. Protein kinase inhibitors against RSK and PKA reversed these increases. Taken together these results support the previous findings that CDK5 acts to attenuate MEK [53], [55], [56], which coordinates with PKA to activate ERK [57]. Additional experiments would fully characterize the role of CDK5 in hepatic glucose regulation, however the current experiments present CDKAL1 and CDK5 into a signaling network that helps to explain their role and can serve as the basis of additional studies.

## Discussion

Recent studies on the genetic contribution to disease have introduced many new genes to discussions of diabetes, cancer and other diseases. These studies are important but difficult to develop into constructive and promising strategies for new therapeutics. There are several types of studies, and each of these has particular challenges. The majority of the recent studies have been genome-wide association studies that monitor the occurrence of atypical distributions single-nucleotide polymorphisms (SNPs) between disease and normal groups, as defined by a phenotype or disease diagnosis. Such discordances are indications of a genetic contribution encoded within the genomic region identified by the particular SNP, defined by the chromosomal region within linkage dysequilbrium of the sentinel SNP (essentially a recombination block). Chief among the difficulties in integrating the recent genetic studies has been their success. A great many studies have been published, often with numerous loci identified, and therefore many candidate genes. The size of the cohort groups insures that the studies are very sensitive, but this also means that many of these findings are of minor impact (effect size), and there has been a sense that since such mutations confer small effects, they may be of minor importance. Since the studies identify genetic loci, the genes involved are inferred by their location within the region of linkage dysequilibrium and therefore are candidates, most of which are not functionally related to the topic of the study. These genes would ultimately be eliminated in functional studies and are not relevant. Modeling such genes in the absence of clearly defined functional roles is counterproductive. Many candidate genes that have been described by these studies are in fact highly novel, giving no real clue as to their role in a biological process. Genomic markers identify candidate genes, but provide no information on the relevant cell type or tissue in which the causative gene exerts its effect. In cases where genes are expressed in more than one cell type, a functional role for the mutant version may exist more than once, and the contribution to a disease could be in one of the cell types or require altered effects in more than one cell type. These challenges are not insignificant, but only really speak to inability to describe a disease solely through genetic data, at least at this time. Instead, a functional assessment is essential to understanding how these recent studies will increase our understanding of human disease and how to treat it.

We have focused on the effect of candidate genes on hepatic glucose regulation as only one of the possible means by which these candidates could play a role in diabetes. Therefore, genes that do not show an effect in this study could be because they are non-involved genomic bystanders, or because their effects could be outside of the liver. Nevertheless, even limited success is still significant, and in fact, we have been able to describe novel and disease-relevant roles for several genes.

### Molecular mechanisms

**Figure 8:**
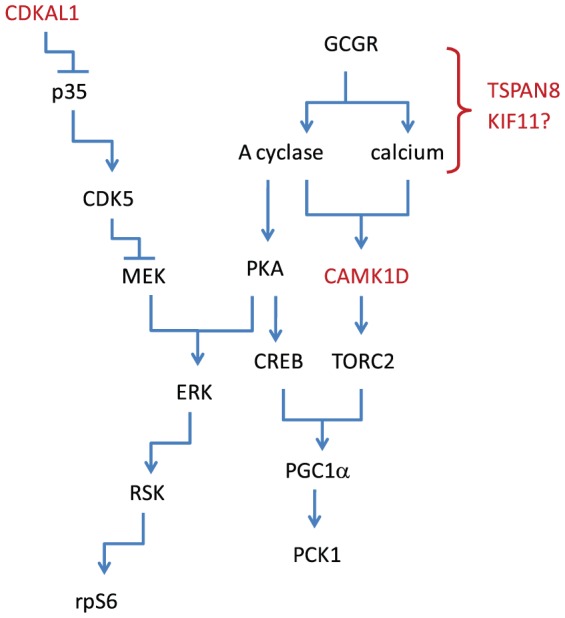
Schematic representation of the role of genes identified in GWA studies on Type 2 Diabetes placed within the context of hepatic glucose regulating signaling pathways. Schematic representation of glucose regulatory signaling pathways in hepatocytes, showing the sites of action of three of the GWA genes for which we have been able to establish a function in hepatocytes. KIF11 could also be included with TSPAN8, but the non-mitotic function of KIF11 is less developed than the functions of the genes indicated in the figure.

This report is focused on placing genes identified in GWA studies into molecular pathways that can help explain which genes within a locus are causally linked to diabetes, and to propose molecular mechanisms that are consistent with their known properties. This report places several genes into physiological and mechanistic roles that help explain how they regulate glucose generally, and by extension, contribute to a genetic predisposition to Type 2 Diabetes, as diagramed in **Figure 8**. That said, additional work will help to definitively characterize these genes and their roles in glucose regulatory signaling pathways.

CAMK1D has been identified as a CAM kinase important for regulating glucose metabolism in hepatocytes, specifically through the regulation of PGC-1 α at the transcriptional level. The evidence suggests that CAMK1D regulates the protein phosphatase calcineurin, as occurs in the regulation of NFAT by calcineurin, through dephosphorylation of NFAT, allowing its translocation into the nucleus. CRTC2 has been shown to be regulated in a similar manner [42]. CAM kinases have been shown to regulate calcineurin through inhibitors of calcineurin, such as Cabin1 [58]. However, detailed molecular studies are important, as the precise mechanism of signaling varies with cell type being studied through the expression of specific regulatory proteins and their localization. One area to follow-up is an examination of activation of CREB and CRTC2. In addition to the activation of calcineurin, CAM kinases can also phosphorylate CREB directly [59]. We were able to observe a high level of activation of CREB in HepG2 cells transduced with a constitutively active CAMK1D kinase (where the calmodulin and negative regulatory domains were deleted), indicating that it is capable of activation CREB. Expression of a kinase inactive form, however, had a very minor effect on CREB levels, indicating that while CAMK1D can activate CREB, it does not appear to be essential for CREB activation (results not shown). As noted by others, methods for assigning roles of CAM kinases through one or two experimental methods is a challenge for CAM kinase signaling research [60], so we note that the data presented here is consistent in its general explanation of CAMK1D in glucose regulation, but await further studies on its molecular mechanism.

The tetraspanins are an emerging family of signal transduction proteins. Although not directly involved in signaling, they play critical roles in the coordination of signaling complexes, including the interaction of GPCRs, such as the glucagon receptor, with adenylyl cyclases and protein kinases [35], [61]. Due to this important role in signal transduction, and the diversity of tetraspanins, with 33 isoforms that interact in patterns specific for the signaling complex and cell type, they present alternatives to the actual membrane receptors as therapeutic targets by antibodies and related technologies for cancer, infection and immune diseases [62], [63]. Therefore it is not surprising that a tetraspanin could be involved in glucagon signaling [64]. TSPAN8, as well as TSPAN proteins co-regulated by TSPAN8 (identified in the transcriptional profling experiments above) could all serve as potential therapeutic strategies for Type 2 Diabetes.

The regulation of PKA/ERK/RSK by CDKAL1/CDK5, as suggested by the experiments in HepG2 cells, is consistent with the effect of CDKAL1 on glyconeogenesis but not on gluconeogenesis. CDK5 is a complex kinase, as it is also regulated by specific interactions with multiple proteins in the cell that coordinate and direct its function [65], [66]. The effect of CDK5 on PKA/ERK/RSK is consistent with both studies on the signaling pathways [52], [53] and on the coordination of these kinases into a signaling complex through AKAP proteins [64], [67]. Demonstrating this role in primary human hepatocytes would be valuable and should be done when protocols for transducing primary hepatocytes without affecting their properties can be developed. The contribution of CDKAL1 and CDK5RAP1 to secretory function and signal transduction continue to emerge as recent studies extend the functions of these proteins [44], [46], [47], [68], [69], but available evidence suggests that these functions are not mutually exclusive.

The finding of KIF11 as a gene that affects glucose regulation was unexpected. The KIF11 gene product, the kinesin Eg5, is the target of monastrol, a potent inhibitor of mitosis and a potential cancer therapeutic [36]. Additionally, the locus for this gene includes IDE, or insulin degrading enzyme, which would be a stronger candidate for a gene affecting diabetes initially. However, subsequent studies have shown that postmitotic cells still express KIF11 and that KIF11 plays additional roles in cellular morphology and structure [70]. In fact, the deletion of the related kinesin KIF1 has been shown to affect insulin secretion in pancreatic cells [71]. Thus, it is possible that KIF11 presents and additional therapeutic strategy for diabetes, although limiting exposure to the liver would be essential. Currently, only unmodified siRNAs are capable of this kind of limited tissue distribution (modified oligonucleotides and vehicle-assisted delivery systems extend the range of target tissues available to oligonucleotide therapeutics).

In conclusion, we have developed a cell-based screen using primary human hepatocytes to investigate the growing list of candidate genes described by recent studies on the human genetic contribution to Type 2 Diabetes. The studies make use of a model system that maintains as much normal human hepatocyte function as is currently possible in vitro, and as such, provides the best experimental evidence that can be obtained in a human cell system. We have identified several genes as having novel roles in hepatic glucose regulation and have been able to define possible mechanisms of action. While additional specific studies are needed to fully validate these as therapeutic targets, these results demonstrate that this system is capable of addressing a difficult challenge to the current human genetic studies of complex diseases, how to evaluate a list of candidate largely uncharacterized genes (as is a common occurrence for many GWA studies) and provide experimentally tractable hypotheses which have the potential to generate therapeutic strategies that are both highly novel and yet explicitly related to the ontology of human disease.

## Supporting Information

Figure S1 **Increased PAS staining in hepatocytes treated with insulin.** Hepatocytes were cultured in low insulin media as indicated. Insulin treatments to stimulate glyconeogenesis were added to cultures 20 hr before fixation and staining. A. Brightfield images of cultures. B. Quanitification of PAS staining levels.
(EPS)

Table S1 **Control genes used in siRNA screening studies.**
(XLSX)

Table S2 **Percent knockdown of target genes by siRNA pools.** mRNA levels as determined by RT-PCR following transfection with target siRNAs.
(DOCX)
