# Supplementary material for: RNAi Screening in Primary Human Hepatocytes of Genes Implicated in Genome-Wide Association Studies for Roles in Type 2 Diabetes Identifies Roles for CAMK1D and CDKAL1, among Others, in Hepatic Glucose Regulation
Source: PLoS One. 2013 Jun 20;8(6):e64946. doi: 10.1371/journal.pone.0064946 (PMC3688709; doi:10.1371/journal.pone.0064946)
Supplement: Table S2 — Percent knockdown of target genes by siRNA pools. mRNA levels as determined by RT-PCR following transfection with target siRNAs. (DOCX) [file pone.0064946.s003.docx]

Supplementary Table s2. Percent knockdown of target genes by siRNA pools.

| Gene | siGenome | On-Target Plus |
| --- | --- | --- |
| ABCC8 | ND | ND |
| ADAMTS9 | ND | ND |
| ALX4 | ND | ND |
| C10Orf7 | 72 | 16 |
| CAMK1D | 78 | 82 |
| CAPN10 | 25 | 23 |
| CDKAL1 | 60 | 46 |
| CDKN2A | ND | ND |
| CDKN2B | ND | ND |
| CREBL2 | 70 | 60 |
| EXT2 | 46 | 57 |
| FLJ32786 | 0 | 0 |
| FTO | 83 | 35 |
| GCK | ND | ND |
| HHEX | 76 | 80 |
| HNF4A | 70 | 67 |
| IAPP | ND | ND |
| IDE | 77 | 83 |
| IGFBP2 | 0 | 0 |
| IPF1 | ND | ND |
| JAZF1 | 78 | 33 |
| KCNJ11 | 3 | 46 |
| KIF11 | ND | ND |
| LGR5 | ND | ND |
| LOC387761 | ND | ND |
| NEUROD1 | ND | ND |
| NOTCH2 | 63 | 76 |
| PPARG | 67 | 60 |
| EXOC4 | 63 | 64 |
| SLC2A2 | 63 | 80 |
| SLC30A8 | ND | ND |
| TCF1 | 61 | 73 |
| TCF2 | 20 | 57 |
| TCF7L2 | 10 | 39 |
| THADA | 47 | 17 |
| TSPAN8 | ND | ND |
| WFS1 | 75 | 87 |

Data is expressed as the percentage knockdown average of three replicates for both the siRNA treated and control samples measured with a probe for the indicated gene , rounded to the nearest integer.

ND: not detected by the probe
